# Evolution of ambiguity tolerance research a scientometric and bibliometric analysis

**DOI:** 10.3389/fpsyg.2024.1356992

**Published:** 2024-06-21

**Authors:** José Rubiales-Núñez, Andres Rubio, Luis Araya-Castillo, Hugo Moraga-Flores

**Affiliations:** ^1^Programa de Derecho y Administración de Empresas, Universidad de Lleida, Lleida, Spain; ^2^Universidad Miguel de Cervantes, Santiago, Chile; ^3^Facultad de Economia y Negocios, Universidad Andres Bello, Santiago, Chile; ^4^Facultad de Psicología, Universidad Diego Portales, Santiago, Chile; ^5^Facultad de Ingeniería y Empresa, Universidad Católica Silva Henríquez, Santiago, Chile; ^6^Facultad de Ciencias Económicas y Administrativas, Universidad de Concepción, Concepción, Chile

**Keywords:** scientometric, ambiguity tolerance, tolerance of ambiguity, Web of Science, bibliometric analysis, decision-making

## Abstract

**Introduction:**

The present study conducts a retrospective bibliometric analysis to examine the quantifiable and qualitative evolution of the concept of tolerance to ambiguity (TA) over time. Additionally, a scientometric analysis using quantitative methods on scientific measurements and trends aims to profile and identify the concept, as well as its development in research themes. The relevance of this study is underscored by the growing interest and development of research on TA, particularly in fields like entrepreneurship where psychological factors are significant.

**Methods:**

The research includes highly relevant literature, such as Budner and Frenkel-Brunswick, which define TA as a predisposition to perceive ambiguous situations as desirable and as a personality variable centered on the emotional and perceptual domain, respectively. Data was obtained from the eight indices comprising the main Web of Science collection, covering research from 1975 to December 2022. A total of 378 articles were identified.

**Results:**

The analysis reveals that scientific production peaked in 2022 with 45 articles. In terms of citations, 7,773 were found, with the highest concentration in 2022, totaling 1,203 citations. This indicates a significant increase in research interest and output related to TA.

**Discussion:**

The study highlights the growing exploration of the concept of TA, emphasizing its importance across multiple disciplines in dealing with uncertainty. The research demonstrates that TA significantly influences decision-making and adaptability, highlighting its value in business and educational settings. By analyzing leading publications, authors, and research centers, the study shows the diversity of approaches to understanding TA, indicating a promising direction for future research.

## Introduction

Ambiguity tolerance (AT) is a concept that is defined as the ability to successfully navigate in doubtful, imprecise, hesitant, unpredictable, and unknown environments (Frenkel-Brunswik, 1949; Budner, 1962a,b). It is theoretically understood as a cognitive factor that directly influences the sensation of restlessness and uncertainty (Frenkel-Brunswik, 1948; Ellsberg, 1961). This concept is crucial for understanding how people adapt to and manage conditions where information is incomplete or unclear.

The first recorded research that named the concept of tolerance of ambiguity was conducted by Frenkel-Brunswik (1948), which has influenced a large number of studies conducted over the last eight decades. She titled her research as “Tolerance toward ambiguity as a personality variable.” In that research, he considered tolerance for ambiguity as a general variable relevant to basic social orientation. The results indicated that tolerant individuals show a greater willingness to accept diversity and ambiguity. This suggests that the willingness to accept these characteristics is more pronounced in people with an attitude of tolerance or judgment. Systematic research with adults and children identified as “liberal” reveals that their perspective transcends national and racial divisions, as well as gender barriers and patterns of dominance-submission. These individuals show openness and flexibility in their worldview. In contrast, prejudiced individuals show rigidity in their cognitive processes. This rigidity is reflected in their inability to consider different perspectives and resistance to modifying deeply held beliefs. There is sensitivity toward statements with qualified terms, in contrast to those without. Furthermore, there is an aversion toward perceptual ambiguity. Prejudiced people prefer rigid stereotypes and lack the propensity to think in terms of probability. They also have difficulties in abandoning pre-established mental sets in intellectual tasks, such as solving mathematical problems.

During the following year, Frenkel-Brunswik (1949) published the article titled “Intolerance of ambiguity as an emotional and perceptual personality variable.” The research focuses on personality, concepts and findings on emotional ambivalence and its expansion in experiments on perceptual ambiguity. This study is based on a project from the University of California Child Welfare Institute, which collected data from 1,500 public school children, ages 11–16, through individual interviews, parent interviews, projective and experimental tests. It was observed that some subjects tolerated emotional ambiguities better. Furthermore, it was concluded that erratic responses indicate general instability in the individual. Intolerance of ambiguity is associated with a lack of flexibility and adaptability. Those who show emotional and social rigidity are less likely to change their minds. It was also observed that intolerant people make fewer spontaneous comments about their childhood, which is revealed in self-reflection and openness to new perspectives.

Along with that, we find Budner (1962a,b) (N.Y. State Psychiatric Institute, page 29), who describes intolerance of ambiguity as the tendency to perceive and interpret ambiguous situations as sources of threat. He describes tolerance of ambiguity as “the tendency to perceive ambiguous situations as desirable.” The study indicates that ambiguous situations are defined by their novelty, complexity and insolubility and are associated with indicators of threat, such as submission, repression, avoidance and operational denial. Tolerance for Ambiguity is related to how these indicators are managed. Furthermore, it is shown that Tolerance of Ambiguity is negatively correlated with conventionality, belief in divine power and attendance at religious meetings. As Tolerance for Ambiguity increases, these aspects decrease.

On the other hand, we find studies on Tolerance of Ambiguity recently carried out around the world. Furthermore, its relationship with multiple variables is observed. These investigations are of great interest for our study, since they contribute to understanding the relationship between tolerance for ambiguity and other relevant variables, providing perspectives that enrich our understanding of the concept. The research conducted by Arquero et al. (2017) with accounting students reveals a negative correlation between Ambiguity Tolerance and communication apprehension, which can be defined as the feeling of anxiety, fear, or insecurity that a person experiences when communicating with others. The findings indicate that as communication apprehension increases, ambiguity tolerance decreases.

In the study by Sokolová and Andreánska (2019) and Sokolova et al. (2019), Ambiguity Tolerance was examined in pre-service teachers and its relationship with the perception of diversity in the classroom. It was found that ambiguity tolerance affects teacher’s classroom effectiveness and management and is positively correlated with their ability to deal with uncertain situations, enhancing their performance in ambiguous work environments. In this analysis, the Ambiguity Tolerance Scale of Multiple Stimulus Types developed by McLain (1993) was employed.

In the article by Rittschof (2016), the relationship between Ambiguity Tolerance and the improvement of measurement systems in the selection of teaching candidates is examined. The Ambiguity Tolerance Scale of McLain (2009) is used, and a positive correlation is found with constructivist orientation in teaching, which can be described as the knowledge actively constructed by the student through their interaction with the environment and the construction of their own meanings. These results indicate a beneficial association between Ambiguity Tolerance and the improvement of teacher measurement systems. Additionally, a scientometric and bibliometric study of this concept could provide deeper insights into its academic impact and the evolving trends in educational research.

In the research conducted by Spinelli et al. (2022), it is shown that Ambiguity Tolerance reflects the ability to handle new, complex, and insoluble situations. Furthermore, this trait is positively associated with greater learning abilities, better intrapersonal behavior, and effective decision-making processes. This indicates that people with high tolerance for ambiguity are better equipped to deal with uncertainty and make informed decisions in challenging environments.

Likewise, in the research conducted by Yang and Xie (2022), which examined 495 Chinese university professors, both men and women, the results revealed that teacher burnout is influenced by Ambiguity Tolerance and enthusiasm. Furthermore, a constructive relationship was found between Tolerance for Ambiguity and enthusiasm, both predictors of teacher burnout (Higher Ambiguity Tolerance and enthusiasm lead to lower burnout).

Finally, in the publication of the research conducted by Yang (2022), we can observe how the variable of Ambiguity Tolerance is associated with emotional intelligence and work engagement, demonstrating a notable increase in articles and citations. This emphasizes the studies on Ambiguity Tolerance, its advancements, relationships, associations, and improvements, to be applied in a multidisciplinary.

Lauriola et al. (2016) research explores the attitude towards ambiguity, revealing three fundamental factors: Discomfort with Ambiguity, Moral Absolutism/Division, and Need for Complexity and Novelty, in samples from Italy and the United States. Furthermore, a second study corroborated these factors through a confirmatory analysis with Italian and English samples, showing that the attitude towards ambiguity incorporates affective, cognitive, and epistemic dimensions. Analyzing tolerance to ambiguity through scientometric and bibliometric methods can provide valuable insights into its academic impact and interdisciplinary relevance.

Hitsuwari and Nomura (2021) developed a Japanese version of the Multidimensional Attitude toward Ambiguity Scale (MAAS), tested on 347 participants, showing good reliability and validity. They revealed significant correlations with other psychological scales and differences between Japanese and Italian participants, suggesting its utility for future cross-cultural research. A comprehensive scientometric and bibliometric study of the concept of tolerance to ambiguity could provide deeper insights into its global relevance and application in various cultural contexts.

Despite having extensive studies on Ambiguity Tolerance, there are no previous researches that allow us to analyze the characteristics of the scientific production on the concept over time, such as bibliometric and/or scientometric analysis. This creates a knowledge gap regarding trends, clusters of currently influential authors, journals, universities, and countries that have published the most.

Given that to date there are no studies on tolerance of ambiguity, it is relevant to address this knowledge gap. This study will allow us to observe and understand how the concept has evolved and progressed, providing an understanding of its development to date and allowing projections on its empirical and theoretical meaning, as well as future lines of research. This is even more important considering that tolerance for ambiguity, according to empirical evidence, has diverse impacts in different organizations and fields today. The impact of this study on ambiguity tolerance is derived from articles published in the Web of Science, a high-impact scientific information network, which contributes to other researchers understanding of the current state of the concept and the field.

Along the same lines, considering the relevance of addressing the current knowledge gap in research on the concept of Tolerance of Ambiguity, this research will provide new information that will contribute to a new knowledge base for researchers to develop their work. Furthermore, this study adopts an advanced and recent practice of scientometric analysis, improving the available knowledge on the methodology used and the temporal evolution of the concept. This method is less biased, rigorous and allows a comprehensive view of scientific research on the concept and its interdisciplinary ramifications.

The objective of this study is to conduct a descriptive analysis of the concept of Ambiguity Tolerance, which will include information from 1975 until December of the year 2022. The study will identify trends related to the number of published articles, authors with the highest number of citations, as well as the most relevant clusters of scientific production, influential articles, journals, countries, and universities with the highest number of publications.

Furthermore, it is advisable that upcoming research endeavors strategically plan, guide, and prioritize future lines of inquiry focusing on exploring the connections between Ambiguity Tolerance and other concepts, as well as its potential implications and outcomes. This approach is particularly pertinent given that Ambiguity Tolerance, as a theoretical concept bolstered by empirical literature, offers significant applicative potential across various domains including organizations, academic institutions, both public and private sector companies, and extensive research settings. This broad applicability underscores its importance in enhancing our understanding and management of uncertainty in diverse environments.

## Materials and methods

The methodology used considered a retrospective bibliometric analysis, which refers to the application of statistical methods to determine the qualitative and quantitative evolution of a scientific research topic, the establishment of publication profiles on the topic, and the identification of trends within a discipline (Diodato, 1994; De Bakker et al., 2005). Additionally, a scientometric analysis was conducted, defined by Nalimov and Mulcjenko (1971) as the development of “quantitative methods of research on the development of science as an informative process. Some of the main topics that scientometrics considers are the ways to measure the quality and impact of research, the understanding of citation processes, the mapping of scientific fields, and the use of indicators in research policy and management (Mingers and Leydesdorff, 2015).

The present study focuses its search on the online database, Web of Science (WoS), which hosts scientific articles from all disciplines. The search is conducted from the earliest records maintained by the database, which correspond to the year 1975, until the latest closed year, 2022, that was current at the time of this research. For broader coverage, we have considered the eight indices that make up the core collection of Web of Science (SSCI, ESCI, SCI-EXPANDED, BKCI-SSH, A&HCI, CPCI-SSH, BKCI-S, CPCI-S).

In this research, we will analyze the most relevant indicators related to the core concept “Ambiguity Tolerance” in all languages. The search yielded 378 articles, which have been cited 7.773 times.

The bibliometric indicators used for the analysis are: articles, citations, journals, institutions, authors, and countries. Additionally, a bibliometric map analysis was conducted with the concept of “Ambiguity Tolerance.” This allows for the design of a detailed map with key concepts based on frequency data and their respective clusters. The results are studied using social network analysis based on graph theory through VOSviewer software version 1.6.15.

The search conducted in the WoS database, updated as of January 5, 2023, is as follows: [TS = (“Ambiguity Tolerance”)] AND DOCUMENT TYPES: (Article) Indexes = SSCI, ESCI, SCI-EXPANDED, BKCI-SSH, A&HCI, CPCI-SSH, BKCI-S, CPCI-S Timespan = 1975–2022. The concept TS refers to the search of the concept in the title, abstract, author keywords, and Keywords Plus of each article in the database.

## Results

### Articles and citations in the study area

**Figure 1:**
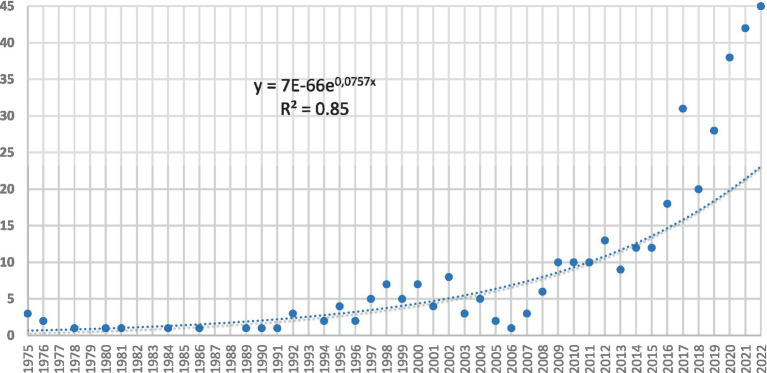
Growth of scientific production. Source: Web of Science data (2023).

After searching for articles related to the concept “Ambiguity Tolerance” between the years 1975 and 2022, a total of 378 articles were identified, spread over the mentioned time frame. These articles received a total of 7.773 citations, with a linear growth described by the equation ART (YEAR) = 7E−66e0,0757 × (YEAR) with an *R*^2^ = 0.85%. Therefore, it can be determined that knowledge production has been accelerating exponentially in the last 5 years, indicating an increase in critical mass in this area of study (see Figure 1).

Figure 1 shows a minimal but steady production of articles until 1992, where it increases until the year 2000, experiencing a small decline that recovers after 2006, and then exhibits sustained growth until 2022, reaching its peak scientific production in the year 2022 with 45 articles. It is worth noting that the last 10 years account for 70.86% of the scientific production, while the last 5 years account for 45.76% of the published articles, reflecting the strong interest that the concept of “Ambiguity Tolerance” has generated in recent years.

**Figure 2:**
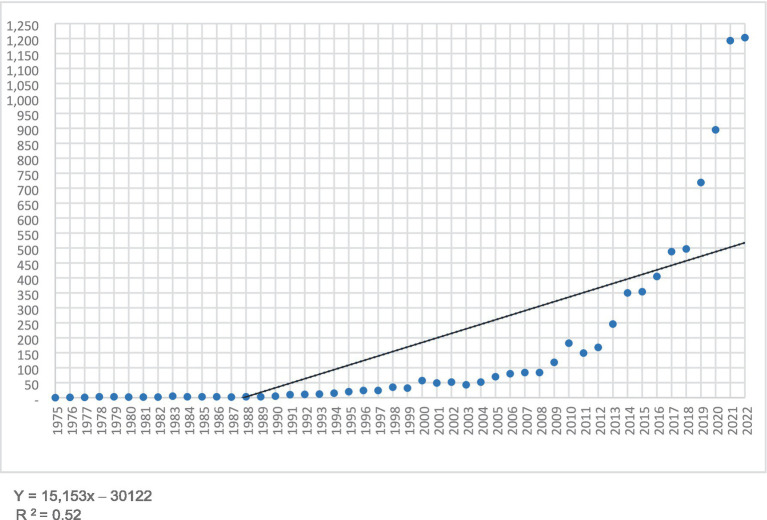
Total number of citations per year. Source: Web of Science data (2023).

In Figure 2, we can observe the number of citations per year for the search concept “Ambiguity Tolerance,” which grows linearly at a rate of 52.47%. The highest number of citations is achieved in the year 2022 with 1.203 citations, followed by the year 2021 with 1.193 citations. It is noteworthy that a high percentage of citations, 58%, is concentrated in the last 5 years, and the last 10 years account for 82%. From this, we can deduce that the last decade has the highest scientific appeal.

Table 1 evaluates the citation rate of the articles. According to the count, there are 7.773 citations in this topic. From the analysis, it can be inferred that 77 articles have not been cited (equivalent to 20.37% of the total), 259 articles have less than 50 citations in WoS (which accounts for 68.52% of the studies conducted), 27 articles have more than 50 but less than 100 citations (equal to 7.14%), 8 articles have more than 100 but less than 150 citations (representing 2.12%), 2 articles have more than 150 citations but less than 200 (representing 0.53%), and finally, 5 articles have more than 200 citations (comprising 1.32% of the studies conducted).

**Table 1:** General structure of citations.

Number of citations	Number of articles	% of articles
More than 200	5	1.32%
More than 150 less than 200	2	0.53%
More than 100 less than 150	8	2.12%
More than 50 less than 100	27	7.14%
Less than 50	259	68.52%
0 citations	77	20.37%
Total	378	100.00%

Source: Own data based on Web of Science (2023).

Regarding the main articles in the set of 378 articles as identified by the WoS database, they are distinguished by the Hirsch index or h-index (Bornmann and Hans-Dieter, 2013). As a general rule, the index favors authors with a long track record who continuously publish works with lasting and above-average impact. Among the articles found, 45 articles surpass 45 citations and, therefore, constitute the most impactful publications in the entire studied set. Among these articles, it is worth noting the one written by Jost (2017), which accounts for 3.67% of the total citations on the topic with 285 citations. This article was published by Political Psychology (Q1) and is affiliated with New York University.

In this article, Jost builds on a previous investigation conducted by Jost et al. (2003a,b), exploring the idea that individuals are rooted in different belief systems that resonate with their psychological needs and interests, including epistemological, existential, and relational needs for certainty, security, and social belonging. Jost et al. (2003a,b), research, based on extensive data, confirms the existence of significant ideological asymmetries based on criteria such as cognitive/perceptual rigidity, personal need for order/structure/resolution, and tolerance for ambiguity/uncertainty, among others.

The second most cited article is authored by Carleton (2012), with 269 citations, accounting for 3.46% of the total citations. It is published in Expert Review of Neurotherapeutics (Q3) by Taylor & Francis LTDA. In this research, the author highlights intolerance of uncertainty as an implicit component in modern anxiety models. Carleton explains that intolerance of uncertainty essentially refers to the fear of the unknown, which has been identified in both normative and pathological samples. Furthermore, the author establishes that recent research has demonstrated that intolerance of uncertainty can be a broad trans-diagnostic dispositional factor for the development and maintenance of clinically significant anxiety.

Table 2 provides details of the 10 most influential articles based on the total number of citations per article, which collectively account for 25.3% of the total citations. These articles show a low concentration of citations for the entire set of articles related to “Ambiguity Tolerance.”

**Table 2:** Articles with the highest citation count in the scientific production.

R	Authors	Year	Title	Journal	TC
1	Jost, John T.	2017	Ideological asymmetries and the essence of political psychology	Political Psychology	285
2	Carleton R, Nicholas	2012	The intolerance of uncertainty construct in the context of anxiety disorders: theoretical and practical perspectives	Expert Review of Neurotherapeutics	269
3	Furnham and Ribchester (1995)	1995	Tolerance of ambiguity: a review of the concept, its measurement and applications	Current Psychology	254
4	Franke et al. (2012)	2012	Experiencing range in an electric vehicle: understanding psychological barriers	Applied Psychology – An International Review-Psychologie Appliquee-Revue Internationale	219
5	Norton (1975)	1975	Measurement of ambiguity tolerance	Journal of Personality Assessment	214
6	Caligiuri, Paula; Tarique, Ibraiz	2012	Dynamic cross-cultural competencies and global leadership effectiveness+D6	Journal of World Business	155
7	Hillen et al. (2017)	2017	Tolerance of uncertainty: conceptual analysis, integrative model, and implications for healthcare	Social Science & Medicine	154
8	Helson, Ravenna M.; Wink, P.	1992	Personality change in women from the early 40s to the early 50s	Academy of Psychology and Aging	141
9	Leary et al. (2017)	2017	Cognitive and interpersonal features of intellectual humility	Personality and Social Psychology Bulletin	140
10	Deyo (2002)	2002	Cascade effects of medical technology	Annual Review of Public Health	132

R, ranking; TC, total citations.

Source: Own elaboration based on Web of Science data (2023).

### Principal authors

Within the set of 378 articles published in the Web of Science database regarding the concept of “Ambiguity Tolerance,” 937 authors are recognized, who have conducted research both as single authors and in co-authorship. There is a high concentration, as evidenced by the percentage of citations held by the most influential authors, which reaches 39.50% of the total citations.

According to the data detailed in Table 3, it is confidently established that the most influential author, considering the number of citations, is Furnham (1994), Furnham and Ribchester (1995), Furnham and Avison (1997), Swami et al. (2018), and Furnham and Treglown (2021) from BI Norwegian Business School, who has published 5 articles related to “Ambiguity Tolerance,” which have been cited 432 times, accounting for 5.55% of the total citations, and holds 3 of these articles among the 45 most influential articles considering the h-index of the search vectors. The second most influential author is John T. Jost from New York University, who with only 1 article related to “Ambiguity Tolerance,” achieves 285 citations and places it among the 45 most influential articles of all time. The details of the other most influential authors of all time on the topic of “Ambiguity Tolerance” are listed in Table 3.

**Table 3:** Most influential authors in “Ambiguity Tolerance.”

R	Author’s name	Institution	TP-AT	TC-AT	%	HA	TP	TC	T45
1	Furnham (1994), Furnham and Ribchester (1995), Furnham and Avison (1997), Swami et al. (2018) and Furnham and Treglown (2021)	BI Norwegian Business School	5	432	5.55%	87	23	36.732	3
2	John T. Jost	New York University	1	285	3.66%	67	187	22.396	1
3	Carleton R. Nicholas	University of Regina	1	269	3.46%	31	65	4.338	1
4	Tracy Ribchester	University of London	1	254	3.26%	3	4	330	1
5	Xu and Tracey (2014, 2015a,b, 2017a,b,c), Xu et al. (2016), Xu and Bhang (2019) and Xu (2020a,b, 2021a,b)	Loyola University Chicago	12	250	3.21%	13	23	540	1
6	Gurel et al. (2010) and Altinay et al., 2012	Oxford Brookes University	2	244	3.13%	34	116	3.474	2
7	Daniele, Roberto	Oxford Brookes University	2	244	3.13%	6	11	343	2
8	Fransiska Buhler	Technische Universitat Chemnitz	1	219	2.81%	8	17	515	1
9	Peter Cocron	Technische Universitat Chemnitz	1	219	2.81%	8	11	508	1
10	Thomas Franke et al. (2012)	Inst Multimdia & Interact Syst	1	219	2.81%	49	136	22.038	1
11	Josef Krems	Technische Universitat Chemnitz	1	219	2.81%	30	132	3.544	1
12	Isabel Neumann	University of Wurzburg	1	219	2.81%	8	18	517	1

R, author ranking; TA-AT, total articles of the author in the search vectors; TC-AT, total citations of the author’s articles in the search vectors; HA, author’s h-index; TP-A, total articles of the author; TC-A, total citations per author; T45, total articles of the author that are among the 45 most influential articles published of all time.

Source: Own elaboration based on Web of Science data (2023).

On the other hand, the number of articles developed and published serves as another metric to determine the contribution of different authors to the generation of knowledge in the field of “Ambiguity Tolerance.” These authors are not always recognized as the most influential, but they are important from the perspective of their contribution to the development of the topic in different scenarios and approaches. For this reason, Table 4 is compiled, detailing those authors who have produced at least 4 articles related to “Ambiguity Tolerance,” indicating the number of articles published on the topic, the total citations of the published articles, the average citations per article, the percentage over the total articles published on the topic, the h-index of the author, total publications, and citations recorded in the WoS platform as of January 2023 by the author.

**Table 4:** Most productive authors in “Ambiguity Tolerance.”

R	Author’s name	University	TP-AT	TC-AT	PC-AT	% Tt	H-A	TP-A	TC-A
1	Xu and Tracey (2014, 2015a,b, 2017a,b,c), Xu et al. (2016), Xu and Bhang (2019) and Xu (2020a,b, 2021a,b)	Loyola University Chicago	12	250	20.83	3.17%	13	23	540
2	Houran and Lange (1996), Houran (1997, 1998a,b,c), Lange and Houran (1999a,b) and Thalbourne and Houran (2000)	ISLA Inst Politcn Gestao & Tecnology	9	194	21.56	2.38%	19	60	1.350
3	Xu and Tracey (2014, 2015a,b, 2017a,b,c) and Xu et al. (2016)	Arizona State University-Tempe	7	196	28.00	1.85%	45	180	6.370
4	Furnham (1994), Furnham and Ribchester (1995), Furnham and Avison (1997), Swami et al. (2018) and Furnham and Treglown (2021)	BI Norwegian Business School	5	432	86.40	1.32%	87	1.247	36.732
5	Hancock and Mattick (2012, 2020), Hancock et al. (2015, 2017) and Hammond et al. (2017)	University of Exeter	5	98	19.60	1.32%	4	11	106
6	Houran and Lange (1996), Lange and Houran (1998, 1999a,b)	Southern Illinois University System	5	111	22.20	1.32%	12	16	627
7	Eley, Diann	The University of Queensland	4	41	10.25	1.06%	25	114	2.282
8	Endres et al. (2009, 2015, 2022) and Endres and Chowdhury (2022)	Eastern Michigan University	4	38	9.50	1.06%	7	10	144
9	Mattick, Karen	University of Exeter	4	86	21.50	1.06%	26	69	1.945
10	Yurtsever, Gulcimen	Istanbul Aydin University	4	54	13.50	1.06%	8	11	109

R, author ranking; TP-AT, total articles of the author considering the search vectors; TC-AT, total citations of the author’s articles in the search vectors; PC-AT, citations per article in the search vectors; % Tt, percentage over the total articles on the search vectors; H-A, author’s h-index; TP-A, total articles of the author; TC-A, total citations of the author.

Source: Own elaboration based on Web of Science data (2023).

From Table 4, it can be observed that there are 10 authors who have successfully published at least 4 articles related to “Ambiguity Tolerance.” It is noteworthy that only 2 of these authors, who are considered the most productive, appear among the most influential in terms of citation count. This demonstrates the heterogeneity in the composition of both authors and their publications. These two influential authors are Adrian Furnham, who has already been mentioned as the most influential, and Hui Xu, who is also included in both analyses.

**Figure 3:**
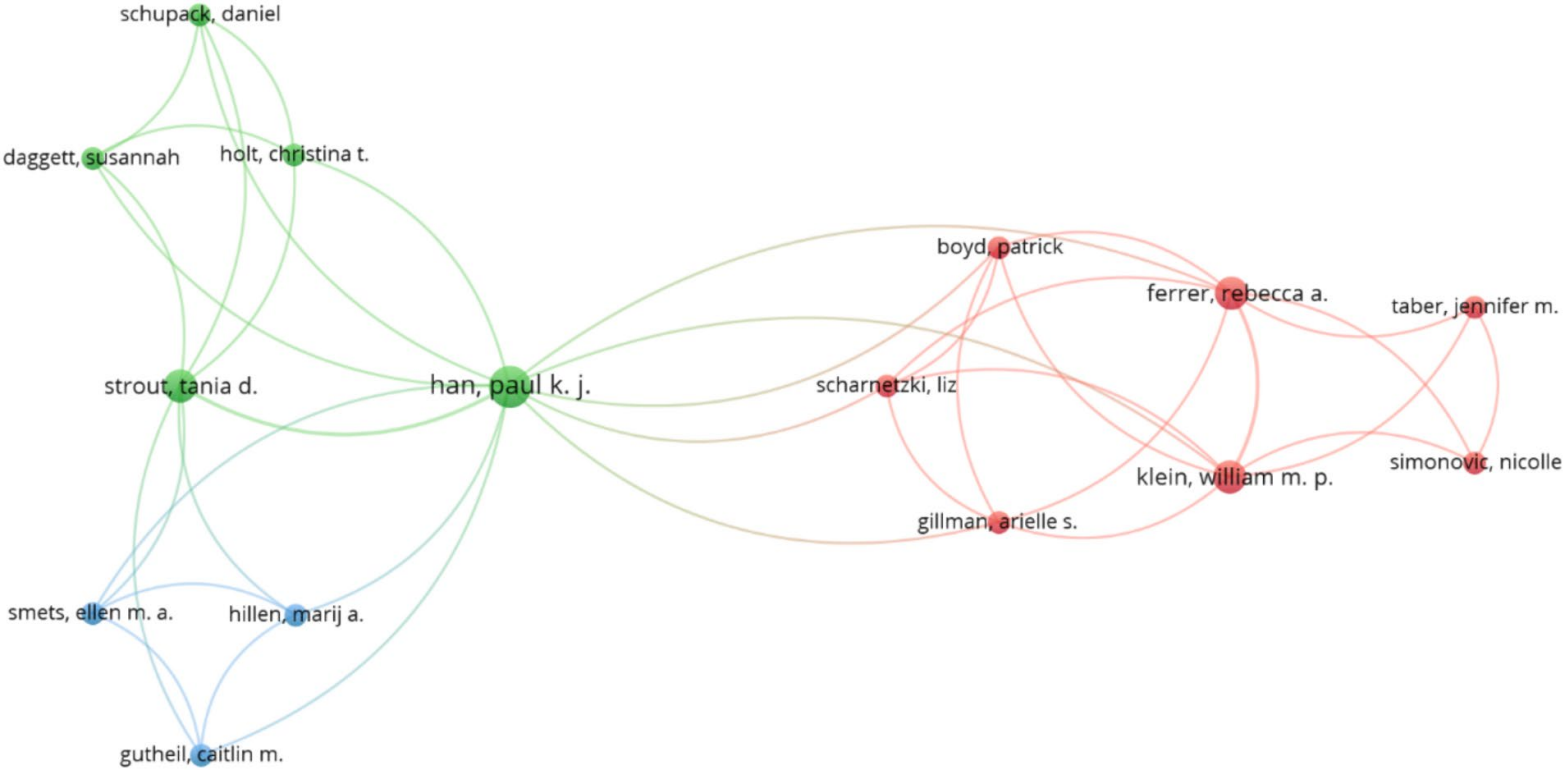
Co-authorship graph for scientific production. Source: Own data created with VOSviewer software.

In relation to the previous paragraphs, Figure 3 presents a graph for analyzing the major co-authorship among authors in relation to the search concepts of “Ambiguity Tolerance.” The articles were input into the VOSviewer software, which groups authors into clusters, resulting in 192 clusters with at least two authors each. Among these clusters, three have been identified as having the most influence on the search concept, and they are detailed in below. Cluster 1 (Red): boyd Patrick, ferrer rebecca a., Gillman et al. (2022), klein, william m. p., scharnetzki liz, Simonovic et al. (2020), taber jennifer m. Cluster 2 (Green): daggett Susannah, Han et al. (2015), holt christina t., schupack Daniel, strout tania d. Cluster 3 (Blue): gutheil caitlin m., Hillen et al. (2017), smets ellen m.

In this way, each cluster represents a group of authors who have collaborated to produce some of the scientific documents. These three clusters are identified in the graph of Figure 3 and highlighted with specific colors.

Cluster 1 is represented in red, and it can be observed that the corresponding circle for Rebecca Ferrer and William Klein is the largest, indicating that they are the authors with the most co-authorships within this cluster. Each of them has 7 co-authorships, meaning they have collaborated with all the authors in their cluster and one author from Cluster 2.

Cluster 2 is identified with green, and in this cluster, the highest, co-authorship belongs to Paul Han, with 13 co-authorships that include all the authors within his cluster and some authors from the other two clusters.

Cluster 3, represented in blue, consists of 3 authors. In this case, all the authors within this cluster are associated only once with all the authors of the same cluster and some authors from Cluster 2. The results suggest a low level of association in researching this topic or, as seen in previous analyses, a high degree of heterogeneity among the authors’ collaborations.

### Principal journals

Regarding the main sources of publication, it is observed that the 378 studied articles have been published in 259 journals indexed in WoS, with a low degree of concentration, as 10 journals have published 80 articles totaling 21.10% of the total publications on the topic, with an average of 21.35 citations per article, totaling 1.708 citations for the set and an h-index of 41. The details of the 10 journals that have published at least 4 articles related to the concepts of “Ambiguity Tolerance” are analyzed in Table 5, with their order determined by the number of published articles and total citations as the second criterion for sorting.

**Table 5:** Web of Science journals where scientific production is generated.

R	Sources (journals)	N	% de Tt	TC-AT	PC-AT	H-AT	FI 5Y	Q
1	Psychological Reports	15	3.96%	259	17.27	7	2.02	Q3
2	Academic Medicine	11	2.90%	314	28.55	7	8.19	Q1
3	Frontiers in Psychology	10	2.64%	61	6.10	3	4.42	Q1
4	Personality and Individual Differences	9	2.38%	387	43.00	8	4.27	Q2
5	BMC Medical Education	8	2.11%	72	9.00	4	3.71	Q2
6	Current Psychology	7	1.85%	277	39.57	2	2.64	Q2
7	International Journal of Psychology	6	1.58%	–	–	–	2.40	Q3
8	Journal of Career Assessment	6	1.58%	87	13.67	5	4.09	Q2
9	International Journal of Production Economics	4	1.05%	170	42.50	3	10.54	Q1
10	Journal of Multilingual and Multicultural Development	4	1.05%	81	20.25	2	2.44	Q2
	Total of the set	80	21.10%	1.708	21.35			

R, ranking; N, total articles considering only the search vector in the journal; % of Tt, percentage of articles over the total articles considering the search vectors; PC-AT, average citations per article in the search vectors; H-AT, h-index considering only the search vectors; TC-AT, total citations considering only the search vectors; FI Y5, journal’s impact factor in the last 5 years; Q, quartile in the category.

Source: Own data based on Web of Science (2023).

Upon analyzing Table 5, it stands out that the most productive journal is “Psychological Reports” from Sage Publications Inc. (United States), which has published 15 articles. However, the most influential journal is “Personality and Individual Differences” from Pergamon-Elsevier Science Ltd., which has the highest number of citations for the set of articles with 387, as well as the highest average citations with 43 citations per article, and also the highest h-index with 8. Finally, the journal “International Journal of Production Economics” holds the highest impact factor in the last 5 years, with a value of 10.540. This impact factor serves as a measure of the quality of these journals. In general, it is not common for these impact indicators to be distributed heterogeneously among the journals, which demonstrates a low degree of concentration in terms of publication sources.

### Institutions

Regarding the main affiliation organizations of the authors, out of the 378 articles, scientists have produced this knowledge with low institutional concentration and are affiliated with 538 organizations. Among these organizations, 10 contribute with at least 7 articles related to the analyzed theme. The details of these institutions are analyzed in Table 6, ordered by their influence in the topic, measured through the number of articles, total citations, average citations, and h-index, in relation to the “Ambiguity Tolerance” search vectors.

**Table 6:** Institutions associated with scientific production based on author affiliation.

R	Organizations	Country	NP	% Tt	TC-AT	PC-AT	h-AT
1	University of London	England	14	3.73%	718	51.29	10
2	Southern Illinois University	United States	10	2.66%	195	19.50	7
3	Arizona State University	United States	9	2.40%	220	24.44	8
4	State University System of Florida	United States	8	2.13%	320	40.00	6
5	Udice French Research Universities	France	8	2.13%	114	14.25	4
6	Universe of Michigan	United States	8	2.13%	445	55.63	6
7	Islamic Azad University	Iran	7	1.86%	5	0.71	2
8	Universite Paris Cite	France	7	1.86%	110	15.71	3
9	University College London	England	7	1.86%	480	68.57	5
10	University of Exeter	England	7	1.86%	184	26.29	5
11	University of Plymouth	England	7	1.86%	160	22.86	5
	Total of the set		92	24.53%	2.951	32.07	61

R, ranking; N, total articles in “Ambiguity Tolerance”; % Tt, percentage of article over the total articles on “Ambiguity Tolerance”; PC-AT, average citations per article for the search vectors; TC-AT, total citations considering only the search vectors; h-AT, h-index considering only the search vectors.

Source: Web of Science data (2022).

From Table 6, it can be deduced that the group of 10 institutions that have published at least 7 articles related to the search concepts accounts for 24.53% of the total articles published on the topic, demonstrating low institutional concentration. Additionally, this group has an h-index of 61, with an average of 32.07 citations per article and a total of 2.951 citations. It is important to consider that some articles, due to co-authorships, may include more than one institution.

We can also establish that the most productive institution is the University of London, with 14 articles, accounting for 3.7% of the total articles. Furthermore, it is the most influential institution considering the number of citations, as it has a total of 718 citations and achieves an h-index of 10. In parallel, we can observe that the University College London, also from England, maintains the highest average citations per article with 68.57 citations on average.

**Figure 4:**
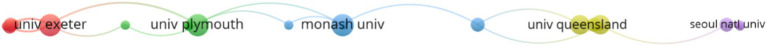
Graph of institutions with the highest co-authorship. Source: Own data processed with VOSviewer software (2023).

Below is a bibliometric analysis of co-authorships among institutions conducting research on “Ambiguity Tolerance.” We identified 55 clusters, each having at least 1 document with 1 citation. Among these 55 clusters, 5 are interconnected and considered the most influential. Details of these groups, Joint Bibliography Clusters for Highly Cited Scientific Production, are provided below, where the institutions with the highest number of co-authorships within each group are highlighted in bold and italics. The graph in Figure 4 shows the connections between different institutions, with different colors representing each of the 5 groups.

Cluster 1 (Red): Coll med vet & life sci, Devon partnership trust, Plymouth univ., Univ Exeter, Univ Glasgow.

Cluster 2 (Green): Cardiff univ., Coventry univ., Univ Plymouth, Wsb univ. Poznan.

Cluster 3 (Blue): Deakin univ., Flinders univ. s Australia, La trobe univ., Monash univ.

Cluster 4 (Yellow): Anthropedia fdn, Univ Minnesota, Univ queensland, Washington univ.

Cluster 5 (Purple): Nanjing univ., Samsung elect, Seoul nati univ.

The graph in Figure 4 displays the 5 clusters with different colors. In the first cluster, the institution that predominates the most is the University of Exeter, which maintains co-authorship with 6 other institutions. In the second cluster, the institution that predominates the most is the University of Plymouth, which also maintains co- authorship with 6 other institutions. In the third cluster, the institution that predominates the most is Monash University, with co-authorship with 4 other institutions. In the fourth cluster, the University of Queensland predominates the most, also with 4 co-authorships. Finally, in the fifth cluster, Seoul National University of San Francisco predominates with 3 co-authorships.

### Countries

In relation to the main countries of affiliation, based on the analysis of the 378 articles, scientists have produced this knowledge with a high geographical concentration, as 41.87% of the articles are concentrated in just 1 country out of a total of 52 countries that have generated at least one article related to the concept of “Ambiguity Tolerance.” Table 7 details the 10 countries that have developed and published more than 10 articles related to the concepts of “Ambiguity Tolerance.” These 10 countries have a combined h-index of 44 with an average of 23.41 citations per article and a total of 7.704 citations.

**Table 7:** Countries associated with scientific production, according to authors’ affiliations.

R	Countries/regions	NP	% Tt	TC-AT	PC-AT	h-AT
1	United States	157	41.86%	4.284	27.29	35
2	England	36	9.60%	1.487	41.31	21
3	Germany	26	6.93%	396	15.23	6
4	Australia	24	6.40%	271	11.29	10
5	People’s Republic of China	24	6.40%	186	7.75	7
6	Iran	16	4.26%	61	3.81	4
7	Canada	13	3.46%	372	28.62	7
8	Israel	12	3.20%	265	22.08	6
9	Spain	11	2.93%	144	13.09	7
10	Turkey	10	2.66%	238	23.80	7
	Summary	329	87.73%	7.704	23.41	44

R, ranking; NP, total articles related to “Ambiguity Tolerance”; % Tt, percentage of articles from the search vectors over the total articles in the same search vectors; TC-AT, total citations considering only the search vectors; PC-AT, average citations per article for the search vectors; h-AT, h-index in “Ambiguity Tolerance.”

Source: Web of Science data (2023).

With the data shown in Table 7, we can confidently conclude that the United States is the most productive country, as it has generated 157 articles related to “Ambiguity Tolerance,” making it the most influential country with the highest number of citations (4.284 citations) and the highest h-index (35). On the other hand, England has the highest average number of citations per article (41.31). With these indicators, the United States maintains a significant lead over the next closest country, which is England, with 36 articles produced, cited 1.487 times, and an h-index of 21.

**Figure 5:**
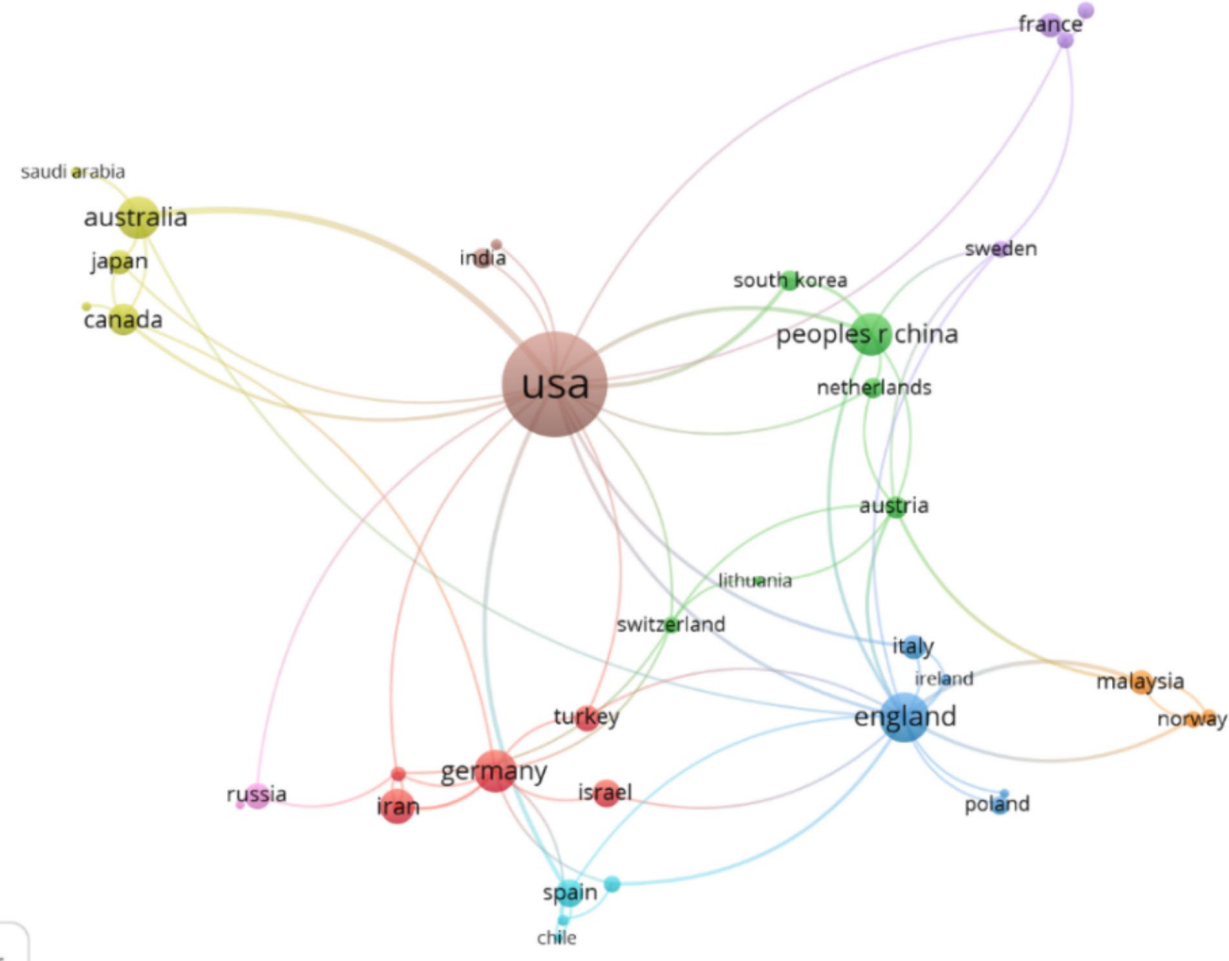
Co-authorship between countries. Source: Own data created with VOSviewer software.

The graph in Figure 5 represents the co-authorship between countries, which shows that 38 out of the 52 countries have at least 1 article with 1 citation in co-authorship. These countries are grouped into 11 clusters, which are detailed in Table 8. For each cluster, the countries that predominate, considering the number of co-authorships with other countries, are marked in bold and italics.

**Table 8:** Co-authorship clusters between countries.

Cluster 1 (Red)	Cluster 2 (Green)	Cluster 3 (Blue)	Cluster 4 (Yellow)
Denmark	Austria	England	Australia
Germany	Lithuania	Ireland	Canada
Iran	Netherlands	Italy	Ghana
Israel	Peoples R China	Poland	Japan
Peru	South Korea	Wales	Saudi Arabia
Turkey	Switzerland		
**Cluster 5 (Purple)**	**Cluster 6 (Sky Blue)**	**Cluster 7 (Orange)**	**Cluster 8 (Gray)**
Belgium	Chile	Bangladesh	Colombia
France	Scotland	Malaysia	India
Sweden	Serbia	Norway	USA
Taiwan	Spain		
**Cluster 9 (Pink)**			
Azerbaijan			
Russia			

Source: Own data based on VOSviewer.

The graph in Figure 5 displays each of the identified clusters represented with different colors, and the size of the circles depends on the number of articles in co-authorships maintained by each country. We can observe the following:

Cluster 1: Germany predominates in this cluster, maintaining co-authorships with 9 other countries.

Cluster 2: China predominates in this cluster, maintaining co-authorships with 6 countries.

Cluster 3: England predominates in this cluster, maintaining co-authorships with 15 countries.

Cluster 4: Australia predominates in this cluster, maintaining co-authorships with 5 countries.

Cluster 5: France predominates in this cluster, maintaining co-authorships with 2 countries.

Cluster 6: Spain predominates in this cluster, maintaining co-authorships with 4 countries.

Cluster 7: Malaysia predominates in this cluster, maintaining co-authorships with 4 countries.

Cluster 8: USA predominates in this cluster, maintaining co-authorships with 17 countries.

Cluster 9: Russia predominates in this cluster, maintaining co-authorships with 3 countries.

### Bibliometric analysis of keywords

**Figure 6:**
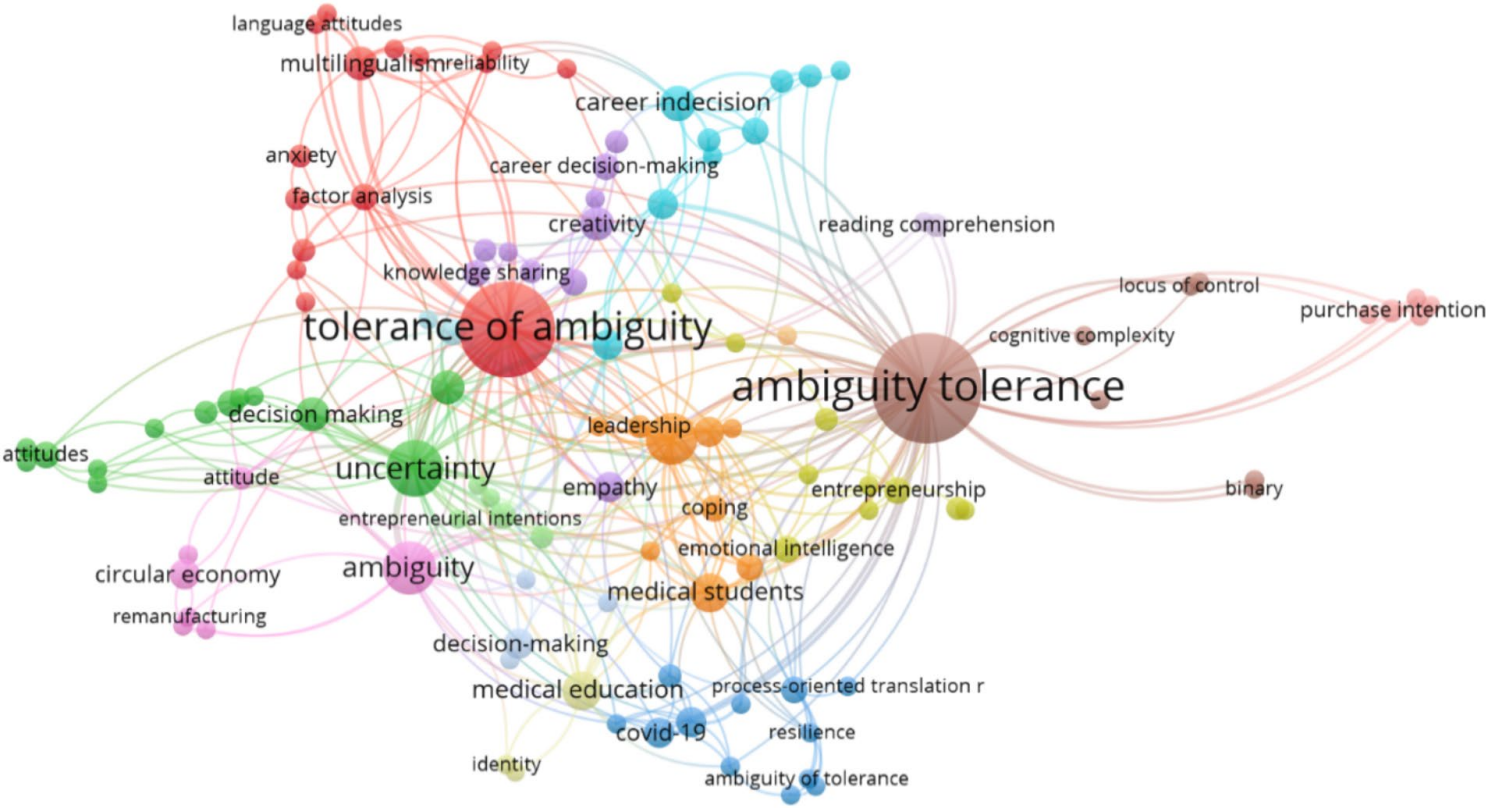
Bibliometric map of research on “Ambiguity Tolerance.” Source: Own data created with VOSviewer software.

Out of the 1.004 author keywords included in the articles published in Web of Science, 114 appear more than 2 times and are used recurrently, as shown in Figure 6. This indicates the presence of 16 clusters, composed as detailed in Table 9, where the most predominant keyword is highlighted in bold and italics.

**Table 9:** Co-occurrence clusters in the author’s use of keywords.

Cluster 1 15 items (Red)	Ambiguity tolerance-intolerance – anxiety – effect size – factor analysis – intolerance of ambiguity – intolerance of uncertainty – language attitudes – linguistic variation – measurement invadias – multilingualism – need for closure – reliability – structural equation model – tolerance of ambiguity – validity
Cluster 2 13 items (Green)	Ambiguity aversion – attitudes – conflicting information – decision making – extraversion – heteronormativity – information processing – neuroticism – personality traits – refugees – self-efficacy – tolerance for ambiguity – uncertainty
Cluster 3 12 items (Blue)	Ambiguity of tolerance – burnout – covid-19 – individual differences – pedagogy – positive psychology – process-oriented trans – qualitative research – resilience – trust – uncertainty tolerance – veterinary students
Cluster 4 10 items (Yellow)	Adolescents – age – big five – career adaptability – career anxiety – emotional intelligence – entrepreneurship – personality characteristics – preference – sensation seeking
Cluster 5 10 items (Purple)	Career decision-making – cognitive flexibility – creativity – empathy – job satisfaction – knowledge management – knowledge sharing – measurement – mind fulness – physicians
Cluster 6 9 items (Sky Blue)	Career counseling – career decision – career decision ambiguity – career decision self-efficacy – career decision-making difficulties – career decision-making self-efficacy – career indecision – path analysis – tolerance – preterence – sensation seeking
Cluster 7 9 items (Orange)	Coping – distress – hermeneutics – leadership – medical students organizational behavic – perfectionism – personality – well-being
Cluster 8 7 items (Gray)	Ambiguity tolerance – binary – cognitive complexity – essentialism – locus of control – magical thinking – occupational stress
Cluster 9 7 items (Pink)	Ambiguity – attitude – circular economy – refurbished products – refurbished products – structural equation modeling – willingness to pay
Cluster 10 5 items (Light Pink)	Bangladesh – confusion avoidance – farmed fish – purchase intention – remanufactured products
Cluster 11 5 items (Dark Green)	Entrepreneurial intention – entrepreneurial intentions – innovativeness – risk propensity – risk-taking
Cluster 12 4 items (Light Blue)	Decision-making – intolerance – mode of delivery – risk readiness
Cluster 13 3 items (Beige)	Identity – medical education – teamwork
Cluster 14 2 items (Lilac)	Foreign language education – reading comprehension
Cluster 15 2 items (Pale Blue)	Experiment – teacher education
Cluster 16 1 item (Light Brown)	Willingness to communicate

Source: Web of Science data (2023).

The graph in Figure 6 reveals a significant number of connections, indicating the level of interconnections between the concepts. However, it is in Table 9 where these concepts are grouped at the cluster level, recognizing the various emphases around which the studied articles are developed. Each cluster in the graph is assigned a specific color for identification. Here are some key observations from the graph:

Cluster 1: The keyword association “tolerance of ambiguity” is the most frequently used with 48 occurrences.

Cluster 2: The word “uncertainty” predominates with 17 occurrences.

Cluster 3: The keyword association “uncertainty tolerance” is predominant with 5 occurrences.

Cluster 4: The words “emotional intelligence” and “entrepreneurship” predominate, each with 4 occurrences.

Cluster 5: The word “creativity” predominates with 6 occurrences.

Cluster 6: The keyword association “career in decisión” is predominant with 7 occurrences.

Cluster 7: The word “personality” predominates with 14 occurrences.

Cluster 8: The keyword association “ambiguity tolerance” is the most prevalent with 65 occurrences.

Cluster 9: The word “ambiguity” predominates with 15 occurrences.

Cluster 10: The word “purchase intention” is the most frequent with 3 occurrences.

Cluster 11: The keyword association “entrepreneurial intention” predominates with 4 occurrences.

Cluster 12: The keyword association “decision making” is the most prevalent with 5 occurrences.

Cluster 13: The keyword association “medical education” predominates with 8 occurrences.

Cluster 14: The keyword association “reading comprehension” predominates with 3 occurrences.

Cluster 15: The word “experiment” predominates with 4 occurrences.

Cluster 16: The keyword association “willingness to communicate” predominates with 2 occurrences.

Along with this, we can see a detailed list of the top 10 author keywords with the highest appearance, ordered from highest to lowest occurrence: Ambiguity Tolerance with 65 occurrences, Tolerance of ambiguity with 47, Uncertainty with 17, Ambiguity with 15, Personality with 14, Medical Students with 8, Medical Education with 8, Career Indecision with 7, Self-Efficacy with 6 and Multilingualism with 6. Which, clearly shows, the words most used by the authors in scientific articles in the line of research tolerance for ambiguity.

## Conclusion and discussion

This research conducted a scientometric and bibliometric analysis of the literature on the concept of Ambiguity Tolerance (AT). Ambiguity Tolerance is a concept that has garnered interest from multiple authors, with greater emphasis in the last decade. This type of analysis provides a solid data foundation using journals indexed in WoS to study the evolution and development of literature on the concept of Ambiguity Tolerance. It is essential to clarify that the primary focus of this study was not to illustrate the scientific production of Ambiguity Tolerance in connection with other variables, but rather to analyze its progression and influence within the academic community independently. This helps isolate the specific impacts and trends associated with Ambiguity Tolerance over time.

As for the published articles, a total of 378 articles were found, reaching its peak production in the year 2022 with a total of 45 articles. Regarding its scientific production, it concentrates 70.86% in the last 10 years and 45.76% in the last 5 years, with a linear growth rate of 85.37% (Figure 1). The findings reveal an upward trend in scientific production related to the concept of Ambiguity Tolerance. There is a constant interest in recent years, suggesting the continuous importance of the topic in the field of research.

As for the Citations, a total of 7.773 were found, reaching its highest point in the year 2022 with 1.203 citations, followed by the year 2021 with a total of 1.193. The concentration of citations is 58% in the last 5 years and 82% in the last 10 years, with a linear growth rate of 52.47% (Figure 2). The examination of citations shows a remarkable increase in recent years. This indicates that the knowledge domain is expanding in terms of the amount of referenced information, implying a strengthening of this knowledge.

With respect to the general citation structure, it was found that 5 articles have more than 200 citations each, followed by 2 articles with more than 150 and less than 200 citations each, in addition to 8 articles with more than 100 and less than 150 citations each, reaching a total of 3.97% of the total citations for the top 15 most cited articles. These articles, which represent a significant percentage of the total citations, clearly demonstrate their great impact and relevance in the academic community. Their high number of citations reflects the influence they have had on research and the interest they have sparked among scholars. These results support the idea that these articles have substantially contributed to the advancement of knowledge in the field of Ambiguity Tolerance study.

Regarding the articles within the most cited scientific production, we have author Jost (2017) with the article titled “Ideological Asymmetries and the Essence of Political Psychology,” which reaches a total of 285 citations, accounting for 3.67% of the total citations. In the second place, we have author Carleton (2012) with his article “The Intolerance of Uncertainty Construct in the Context of Anxiety Disorders: Theoretical and Practical Perspectives,” which has 269 citations and accounts for 3.46% of the total citations. In the third place, we have the article “Tolerance of Ambiguity: A Review of the Concept, Its Measurement, and Applications,” authored by Furnham and Ribchester (1995), with a total of 254 citations and 3.26% of the total citations. Together, the top 3 most cited articles reach 808 citations and 10.39% of the total citations. These studies highlight the broad recognition and significant influence they have exerted in the field of Ambiguity Tolerance, significantly driving the progress of knowledge in this area of research. Their impact has been fundamental for the advancement and development of the understanding of Ambiguity Tolerance, providing a solid foundation and contributing significantly to the existing body of knowledge. Moreover, they have generated an important starting point for future research and stimulated the exploration of new perspectives and approaches in the study of ambiguity and its tolerance.

As for the most productive authors in “Ambiguity Tolerance,” a total of 937 authors were identified, among whom Xu Hui from “Loyola University Chicago” stands out with 12 Xu and Tracey (2014, 2015a,b, 2017a,b,c), Xu et al. (2016), Xu and Bhang (2019), and Xu (2020a,b, 2021a,b) published articles and a total of 250 citations. Followed by author James Houran from “ISLA Inst Politcn Gestao & Technology” with 9 Houran (1997, 1998a,b,c), Lange and Houran (1999a,b) and Thalbourne and Houran (2000) published articles and a total of 194 citations, and in third place, author Terence Tracey from “Arizona State University-Tempe” with 7 Xu and Tracey (2014, 2015a,b, 2017a,b,c) and Xu et al. (2016) published articles and a total of 196 citations. The obtained results highlight the outstanding contribution of the most productive authors in the field of Ambiguity Tolerance. Their prolific research and significant number of citations received clearly reflect their importance and recognition in the area. Their work has been instrumental in advancing the understanding of Ambiguity Tolerance and has made a significant impact on the academic community.

Regarding the main journals that have generated the highest scientific production, we find “Psychological Reports” in the first place with a total of 15 articles, followed by the journal “Academic Medicine” with a total of 11 published articles, in the third place is “Frontiers In Psychology” with a total of 10 publications, and concluding with the journals “Personality And Individual Differences” and “BMC Medical Education,” with 9 and 8 publications, respectively. These results highlight the top 3 distinguished journals in generating academic research in the field, characterized by their outstanding contribution and continuous publication of quality scientific articles. These journals have played a fundamental role in promoting and disseminating research on Ambiguity Tolerance.

With regard to the institutions associated with the highest scientific production, based on author affiliations, a total of 538 organizations were identified. In the first place, we have the “University of London” from England, with a total of 14 published articles. In the second place, we have the “Southern Illinois University” from the United States with 10 articles, and in the third place, we have the “Arizona State University” from the United States with 9 published articles. Rounding off the ranking with 8 published articles are the “State University System of Florida” and the “University of Michigan,” both from the United States, and the “Udice French Research Universities” from France. The results highlight the most relevant institutions in generating scientific knowledge. These institutions stand out for their remarkable contribution to academic research in the field, playing a prominent role in advancing the understanding of the concept of Ambiguity Tolerance. Their significant contribution has made a notable impact on the development and progress in this important and relevant area, generating scientific knowledge that can be applied to various research and applications in the context of its applicability in companies and organizations.

Upon examining the countries associated with the highest scientific production based on author affiliations, the United States stands out as the clear leader in the field of the concept of Ambiguity Tolerance, with an outstanding total of 157 published articles. At a considerable distance, we find England with 36 articles, followed by Germany with 26, and in a tie, China and Australia with 24 articles each. The analysis of scientific production reveals a clear landscape: the United States positions itself as the undisputed leader in generating knowledge on the concept of Ambiguity Tolerance. These countries demonstrate their commitment to research and their prominent role in advancing it. Their significant contributions have strengthened the field of study on the concept of Ambiguity Tolerance and have laid the groundwork for future developments and applications.

Regarding the clusters with the highest co-authorship association in scientific production, a total of 192 clusters were identified. Among them, the two clusters with the highest number of co-authorships are the authors Rebecca Ferrer and William Klein, who have collaborated on seven occasions. In the second cluster, the most prominent co-authorship belongs to Paul Han, with an impressive total of 13 collaborations. Additionally, it is observed that the institutions “University of Exeter” and “University of Plymouth” are the ones that predominate in terms of co-authorships, with a total of six collaborations with other institutions. Examining the co-authorship clusters in scientific production highlights the participation of these outstanding authors, who have maintained a constant production of publications over time. This demonstrates a lasting impact and above-average productivity, further reinforcing the relevance of their work in the field of Ambiguity Tolerance.

The information about the co-authorship clusters between countries and the co-occurrence of keywords provided valuable insights into the global collaboration in the field of Ambiguity Tolerance. The presence of 9 co-authorship clusters involving countries like Germany, China, England, Australia, France, Spain, Malaysia, the United States, and Russia indicates a robust network of international scientific collaboration. This network reflects the diversity and knowledge exchange that occurs at a global level, fostering significant advancements in the scientific field. Furthermore, the co-occurrence of the keywords “Ambiguity Tolerance” and “Tolerance of Ambiguity” in the co-authorship clusters highlights the interconnectedness and shared focus on these concepts within the scientific community. The usage of these keywords signifies a common language and understanding of the research topic, facilitating communication and cooperation among researchers from different countries and institutions. Overall, the findings from the co-authorship clusters between countries and the co-occurrence of keywords underscore the global relevance and impact of research on Ambiguity Tolerance. The collaborative efforts across borders and the alignment of research interests contribute to a deeper understanding of the concept and its implications in various fields and contexts.

Among the articles that have received the greatest number of citations, various investigations stand out, which are distinguished by their ability to significantly enrich and improve readers’ understanding of the concept of tolerance for ambiguity. Caligiuri and Tarique (2012) demonstrates that the intersection of open personalities and deep intercultural experiences in global leaders significantly fosters ambiguity tolerance, an essential pillar for effective global leadership. This finding highlights the critical importance of ambiguity tolerance, influencing favorable supervisor evaluations and marking a clear path for the strategic development of competent global leaders. The research conducted by Helson and Wink (1992) highlighted a marked increase in ambiguity tolerance among women aged 40–50 years, demonstrating significant progress in their ability to manage uncertainties with less stress and emphasizing the vital importance of cognitive flexibility for emotional well-being in middle age. Leary et al. (2017) research investigated intellectual humility, highlighting its connection with tolerance for ambiguity. Individuals with greater intellectual humility, defined as recognition of the potential fallibility of one’s beliefs, were found to demonstrate greater tolerance for ambiguity. This suggests that intellectual humility facilitates openness and flexibility in uncertain situations, allowing individuals to better handle ambiguity and be more open to revising their beliefs in light of new information. This link underscores the importance of intellectual humility for cognitive adaptability and openness to diverse perspectives.

The limitations of this study are mainly related to its methodological approach (bibliometric and scientometric analysis). Although the inclusion criterion was established for scientific documents indexed in Web of Science, obtained from the eight indices that make up the core collection of Web of Science (SSCI, ESCI, SCI-EXPANDED, BKCI-SSH, A&HCI, CPCI- SSH, BKCI-S, CPCI-S), other quality control filters for the articles were not taken into account.

For future research, it would be relevant to consider the adoption of supplementary measures to assess the quality and reliability of the selected documents in order to strengthen the robustness of the study. In addition to the exclusive inclusion of articles from WoS databases, it is important to acknowledge that access has been restricted by not considering other sources of information.

Furthermore, it was not feasible to conduct a more precise control over the content of the analyzed articles, although this is due to the preliminary nature of the article, whose objective is to provide an overview of the scientific production associated with the concept of ambiguity tolerance.

In future research, it would be relevant to consider adopting additional measures to assess the quality and reliability of the selected documents, with the aim of further strengthening the study’s robustness.

By including articles solely from the WoS databases, the scope of access will be limited, as other relevant sources of information will be excluded. Additionally, a more thorough content control of the analyzed articles could not be conducted due to the introductory focus of this article, which aims to provide an overview of the scientific production related to the concept of ambiguity tolerance.

Absolutely, taking into account the limitations of this research is crucial to provide further contributions in this field. It would be feasible and beneficial to expand and conduct additional analyses by considering articles not included in the WoS database. This approach would open new perspectives and enrich the understanding of the field of study. By exploring other sources of information and conducting more comprehensive content analyses, researchers can gain a deeper insight into the concept of ambiguity tolerance and its related research. This will not only enhance the current understanding but also pave the way for new avenues of exploration and discovery.

Indeed, the suggestions derived from this study highlight the relevance of ambiguity tolerance not only in the realm of psychology but also in the business domain, encompassing both research and professional practice, among other important aspects. Based on the findings of this research, there are promising perspectives for future lines of investigation that hold potential applications in a wide range of organizations, institutions, public and private companies, as well as in research and academic settings. It is crucial for workplaces to consider this aspect in their activities, in order to fully harness the value of ambiguity tolerance in all its dimensions.

By integrating the concept of ambiguity tolerance into organizational practices, leaders and managers can promote a culture that embraces uncertainty and fosters adaptability. Emphasizing tolerance for ambiguity can enhance problem-solving abilities, decision-making processes, and innovative thinking within the workforce. Additionally, recognizing the importance of ambiguity tolerance in various contexts may lead to the development of training programs and interventions that cultivate this trait in individuals and teams, ultimately contributing to improved performance and resilience in dynamic environments.

Furthermore, as ambiguity tolerance intersects with other psychological constructs, such as emotional intelligence and job engagement, future research could explore the intricate relationships between these variables, providing valuable insights into the complex dynamics that influence employee well-being and job satisfaction.

In academia, understanding ambiguity tolerance can enrich the pedagogical approaches used by educators. By acknowledging individual differences in tolerance for ambiguity among students, instructors can tailor their teaching strategies to accommodate diverse learning styles and create an environment that encourages curiosity, exploration, and critical thinking.

Overall, the findings of this study serve as a solid foundation for future investigations that can significantly impact organizational and educational practices. Integrating the concept of ambiguity tolerance into various domains can lead to more adaptive, innovative, and successful endeavors, thereby enhancing the capabilities of individuals and delivering substantial benefits to society at large. This integration not only encourages resilience but also cultivates a culture of continuous improvement and problem-solving.

## Data availability statement

The original contributions presented in the study are included in the article/Supplementary material, further inquiries can be directed to the corresponding author.

## Author contributions

JR-N: Conceptualization, Data curation, Formal analysis, Funding acquisition, Investigation, Methodology, Project administration, Resources, Software, Supervision, Validation, Visualization, Writing – original draft, Writing – review & editing. AR: Conceptualization, Data curation, Formal analysis, Funding acquisition, Methodology, Supervision, Validation, Writing – original draft, Writing – review & editing. LA-C: Conceptualization, Funding acquisition, Methodology, Supervision, Validation, Writing – original draft, Writing – review & editing. HM-F: Data curation, Investigation, Software, Writing – original draft, Writing – review & editing.
